# Clinical utility of serum HER2/neu in monitoring and prediction of progression-free survival in metastatic breast cancer patients treated with trastuzumab-based therapies

**DOI:** 10.1186/bcr1020

**Published:** 2005-04-08

**Authors:** Francisco J Esteva, Carol D Cheli, Herbert Fritsche, Monica Fornier, Dennis Slamon, Robert P Thiel, Diana Luftner, Farooq Ghani

**Affiliations:** 1 The University of Texas, MD Anderson Cancer Center, Houston, TX, USA; 2 Bayer HealthCare, LLC, Diagnostics Division, Tarrytown, NY, USA; 3 Memorial Sloan Kettering Cancer Center, New York, NY, USA; 4 University of California, Los Angeles, Department of Medicine, Los Angeles, CA, USA; 5 Thiel Statistical Consultants, Oxford, CT, USA; 6 Charité Hospital, Universitätsmedizin Berlin, Berlin, Germany

## Abstract

**Introduction:**

The purpose of this retrospective study was to determine the clinical utility of serum HER2/neu in monitoring metastatic breast cancer patients undergoing trastuzumab-based therapy and to compare these results with those obtained using cancer antigen (CA) 15-3. We also sought to determine whether early changes in serum HER2/neu concentrations could be a predictor of progression-free survival.

**Methods:**

Sera were obtained retrospectively from 103 women at four medical institutions. Patients eligible for participation were women with metastatic breast cancer who had HER2/neu tissue overexpression and were scheduled to be treated with trastuzumab with or without additional therapies as per the established practices of the treating physicians. A baseline serum sample for each patient was taken before trastuzumab-based therapy was started. Patients were subsequently monitored over 12 to 20 months and serum samples were taken at the time of clinical assessment and tested with Bayer's HER2/neu and CA15-3 assays.

**Results:**

Concordance between clinical status in patients undergoing trastuzumab-based treatment and HER2/neu and CA15-3 used as single tests was 0.793 and 0.627, respectively, and increased to 0.829 when the tests were used in combination. Progression-free survival times did not differ significantly in patients with elevated baseline HER2/neu concentrations (≥ 15 ng/mL) and those with normal concentrations (<15 ng/mL). However, progression-free survival differed significantly (*P *= 0.043) according to whether the patient's HER2/neu concentration at 2 to 4 weeks after the start of therapy was >77% or ≤ 77% of her baseline concentration. The median progression-free survival times for these two groups were 217 and 587 days, respectively. A similar trend was observed for a subcohort of patients treated specifically with a combination of trastuzumab and taxane.

**Conclusion:**

These findings indicate that serum HER2/neu testing is clinically valuable in monitoring metastatic breast cancer patients undergoing trastuzumab-based treatment and provides additional value over the commonly used CA15-3 test. The percentage of baseline HER2/neu concentrations in the early weeks after the start of therapy may be an early predictor of progression-free-survival.

## Introduction

The human-epidermal-growth-factor receptor 2 (HER2, also known as neu, ErbB-2, and p185^HER2^) is a transmembrane glycoprotein with an intracellular tyrosine kinase activity and an extracellular domain very similar to those of the epidermal-growth-factor-binding domain of the epidermal-growth-factor receptor [1]. The *HER2/neu *proto-oncogene is amplified and/or overexpressed in approximately 20 to 25% of invasive breast cancers [2,3]. HER2/neu overexpression has been associated with a poor rate of disease-free survival [4]. The role of HER2/neu as a predictive marker of response to hormone therapy and chemotherapy is controversial [5-8]. The extracellular domain (ECD) of the HER2/neu protein is frequently cleaved and released into the circulation, where it can be detected by ELISA in up to 45% of patients with metastatic breast cancer [9]. Rising serum HER2/neu concentrations have been associated with progressive metastatic disease and poor response to chemotherapy and hormonal therapy [10]. Lipton and colleagues [11] showed that patients with an elevated HER2/neu ECD before therapy were less likely to respond to second-line endocrine therapy. The same team evaluated the predictive role of serum HER2/neu ECD in a randomized clinical trial of tamoxifen versus letrozole for patients with metastatic breast cancer. In that trial, patients with low concentrations of circulating HER2/neu ECD had improved response rates and time to progression of disease if they had been treated with letrozole. In patients with an elevated HER2/neu ECD, however, there were no significant differences in outcome between patients treated with tamoxifen and those treated with letrozole [12].

Trastuzumab (Herceptin™, Genentech, South San Francisco, CA, USA) is the only HER2/neu-directed therapy approved by the Food and Drug Administration (FDA) for the treatment of patients with metastatic breast cancer. Trastuzumab is a humanized monoclonal antibody directed against the HER2/neu ECD. Single-agent response rates range from 12 to 30%, depending on the HER2/neu status of the tumor and the patient's prior treatment [13,14]. Response rates and time to disease progression in patients with metastatic breast cancer are better when trastuzumab is combined with chemotherapy than when treatment is with chemotherapy alone [15]. Trastuzumab has been shown to be synergistic with a variety of commonly used chemotherapies such as paclitaxel, docetaxel, platinum salts, and vinorelbine [15-17].

The most commonly used methods of selecting patients for trastuzumab monoclonal antibody therapy are immunohistochemistry and fluorescence *in situ *hybridization (FISH) [18]. Retrospective studies have shown that *HER2/neu *gene amplification, measured using FISH, is the best predictive marker of response to trastuzumab-based therapy [14]. The role of circulating HER2/neu ECD as a predictive marker of response to such therapy and its role for monitoring therapy in metastatic breast cancer are not well defined. For the current retrospective, multicenter evaluation, we sought to determine the clinical utility of serum HER2/neu in monitoring metastatic breast cancer patients undergoing trastuzumab-based therapies and to compare these results with those obtained using CA (cancer antigen) 15-3. Additionally, we sought to determine whether early changes in serum HER2/neu concentrations can be a predictor of progression-free survival.

## Materials and methods

### Patient population

Sera were obtained from 103 subjects meeting the study entry criteria at four medical institutions: the University of Texas MD Anderson Cancer Center, Memorial Sloan Kettering Cancer Center, the Charité Hospital, and the University of California. The study was reviewed and approved at each site by the local institutional review board and written informed consent was obtained from each enrolled subject. Patients eligible for participation were women with metastatic breast cancer who had HER2/neu overexpression as determined by immunohistochemistry (scores of 2+ or 3+ on the Dako Herceptest, Dako Corp, Carpinteria, CA, USA, or CB11 antibody Pathway, Ventana Medical Systems Inc, Tucson, AZ, USA) or by FISH (PathVysion HER2/neu method, Vysis Inc, Downers Grove, IL, USA) or the INFORM HER2/neu gene detection system, Ventana Medical Systems) and who were scheduled to be treated with trastuzumab with or without additional therapies as per the established practices of the treating physicians. Patients were trastuzumab-naive at entry to the study. The first (baseline) serum sample for each patient was taken before trastuzumab-based therapy was started. Patients were subsequently monitored over 12 to 20 months during trastuzumab-based therapy, with at least four subsequent serial serum samples being collected at least 4 weeks apart, at the discretion of the physician at the time of clinical assessment.

### Evaluation of tumor response

Clinical status was determined by available clinical data in the form of physical examination and imaging techniques (ultrasound, x-ray, computed tomography, magnetic resonance imaging) at each patient visit. All patients had documented, measurable metastatic breast cancer at entry to the study. Response to treatment was assessed according to the criteria of the World Health Organization [19] or RECIST (response evaluation criteria in solid tumors) [20]. Clinical status – whether response was complete or partial and whether disease was stable or progressive – was determined at each patient visit. Complete or partial responses were confirmed at a minimum of 4 weeks after complete or partial response was observed.

### Sample collection and serum HER2/neu antigen testing

Serum samples were processed immediately upon collection of blood and kept at -20°C or colder. Serum samples were either tested at the site or were subsequently shipped on dry ice to Bayer Diagnostics (Tarrytown, NY, USA) for batch testing. Frozen samples were tested immediately upon initial thawing.

The HER2/neu antigen testing was performed using either the Bayer Immuno1 or ADVIA Centaur™ automated assays. Both methods are currently cleared by the FDA with an indication for follow-up and monitoring of patients with metastatic breast cancer. Previous studies have shown similar diagnostic performance of the automated methods, with very high correlation between the methods (*r*^2 ^= 0.99) (product method sheet), because the antibodies used for capture and detection of the circulating HER2/neu antigen are identical for these methods. A serum HER2/neu concentration of 15 ng/ml has been defined as the upper limit of normal [21].

The Immuno1 assay technology has been previously described [21]. Briefly, the ADVIA Centaur HER2/neu assay is a two-site sandwich immunoassay using direct, chemiluminescent technology. The Lite Reagent is composed of the monoclonal mouse antibody TA-1 labelled with acridinium ester. The Fluorescein Conjugate Reagent is composed of the monoclonal mouse antibody NB-3 labelled with fluorescein. These two monoclonal antibodies are specific for unique epitopes on the extracellular domain of HER2/neu. The Solid Phase is composed of purified monoclonal mouse capture antibody (antifluorescein) covalently coupled to paramagnetic particles, which binds the immunocomplex. The reaction is initiated and the measured chemiluminescence is directly proportional to the quantity of HER2/neu antigen in the sample.

Samples were also tested for CA15-3 concentrations using the ADVIA Centaur automated assay in accordance with the manufacturer's instructions. Single determinations of HER2/neu and CA15-3 were obtained on each specimen tested.

### Statistical analysis

#### Monitoring of metastatic breast cancer patients with serum HER2/neu

To determine the association between HER2/neu and disease status throughout the monitoring period, patients with stable partial or complete response were grouped together and considered as having no disease progression. This group was compared with the patients whose disease was progressing. For each pair of serial measurements, changes in HER2/neu of ≥ 15% or < 15% and in CA15-3 of ≥ 21% or <21% were considered to indicate progression or lack of progression, respectively. These values (i.e., the 15% or 21% change) were based on the variability of each marker when tested longitudinally over time in a population of apparently healthy woman (product method sheets). Concordance with clinical status was determined for both markers with 95% confidence intervals (CI) as single tests and, when the two markers were used in combination, as a series test. For a series test, change was considered positive if it was ≥ 15% for HER2/neu ***and ***≥ 21% for CA15-3. All other changes were considered no change. Concordance estimates and their CIs were obtained using Windows Excel with the Resampling Stat add-in.

#### Association between baseline HER2/neu concentrations and progression-free survival

To determine whether the baseline concentration of serum HER2/neu is a predictor of progression-free survival in patients with metastatic breast cancer treated with trastuzumab-based therapies, time to progression was measured from baseline to the date of first documented disease progression for the serum-positive patient group (pretreatment HER2/neu ≥ 15 ng/ml) as compared with the serum-negative patient group (pretreatment HER2/neu <15 ng/ml), using the Kaplan–Meier method. A log-rank test was used to determine if significant difference in progression-free-survival existed between the two groups.

#### Association between percentage of baseline HER2/neu concentrations after 2 to 4 weeks of therapy and progression-free survival

Where possible based on serum sample availability, the percentage of baseline HER2/neu concentrations at 2 to 4 weeks of therapy was determined for a patient. ROC (receiver operating characteristic) curve analysis as a function of the percentage of change in the HER2/neu concentrations between baseline and 2 to 4 weeks after initiation of therapy was used to determine the optimal cutoff point. Progression-free survival was compared between the groups of patients above and below the cutoff point using the Kaplan–Meier method. A log-rank test was used to determine significant difference in progression-free-survival between the two groups.

Kaplan–Meier analysis and graphs were generated using SPSS (Statistical Package for the Social Sciences) for Windows (version 11.0).

## Results

This retrospective study included a total of 103 women with HER2/neu-overexpressing metastatic breast cancer treated with trastuzumab-based therapies. The mean serum HER2/neu concentration at baseline was 170.3 ng/mL, with a median of 24.0 ng/mL. The mean HER2/neu value for patients with an immunohistochemistry score of ≤ 2+ was 182.8 ng/mL. For patients with an immunohistochemistry score of 3+, the mean Her-2/neu value was 201.8 ng/mL; a two-sample *t*-test indicated that the difference was not significant (*t *= 0.118, *df *= 74, *P *= 0.9). The mean serum CA15-3 concentration at baseline was 224.4 ng/mL, with a median of 42.6 ng/mL. The average age of the patients at entry to the study was 54 years (range 26 to 97 years). Table 1 shows the characteristics of the patients with respect to grade of HER2/neu overexpression and treatments.

To assess the clinical value of serum HER2/neu in monitoring patients longitudinally over the time course of trastuzumab-based treatment, a total of 99 of the evaluable patients with both HER2/neu and CA15-3 measurements were evaluated. These patients generated a total of 362 visit pairs for analysis. The median of time points was 4. Table 2 shows the estimates of concordance with clinical status for each method alone and in combination as a series test. HER2/neu showed considerably greater concordance than CA15-3 in patients monitored longitudinally, and when the two tests were used in combination, the concordance with clinical status was slightly better than when HER2/neu was used alone.

Baseline concentrations of serum HER2/neu were elevated (that is, ≥ 15 ng/mL) in 70% (72) of the 99 evaluable patients. Of the 27 patients with a normal HER2/neu baseline value, disease progressed in 21, and Her-2/neu values were available for 20 of these at the time when progression was found: 7 (35%) had elevated HER2/neu values at that time. Of the 27 patients with normal baseline values of Her-2/neu, 10 who were receiving the trastuzumab–taxane combination had progression of their disease and 3 of these 10 had elevated HER2/neu values at the time of the progression.

Kaplan–Meier analysis in patients with elevated baseline concentrations of HER2/neu (≥ 15 ng/mL) versus those with normal baseline concentrations (<15 ng/mL) showed no significant differences in progression-free survival between these two groups (log-rank statistic = 2.38, *df *= 1, *P *> 0.05). The median follow-up was 237 days. The median progression-free survival times were 324 days for patients with normal baseline values of HER2/neu and 462 days for those with elevated baseline values.

**Figure 1:**
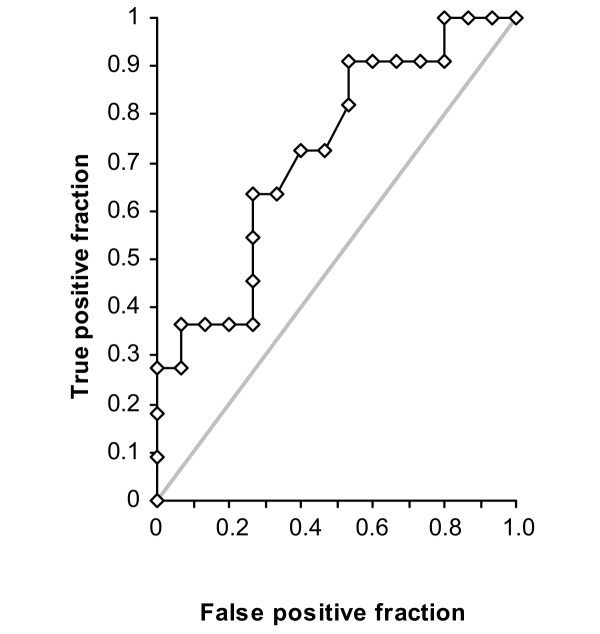
ROC curve analysis for progression of metastatic breast cancer in 26 patients. Nineteen of these patients were treated with trastuzumab (Herceptin) in combination with a taxane, one with trastuzumab (Herceptin) in combination with vinorelbine, and six with single-agent Herceptin without chemotherapy. The curve shows disease progression as a function of the percentage of change in the HER2/neu concentration from baseline to 2 to 4 weeks after the start of therapy. HER, human-epidermal-growth-factor receptor; ROC, receiver operating characteristic.

We determined the association between percentage of baseline HER2/neu concentrations at 2 to 4 weeks after the start of therapy and progression-free survival in a subcohort of 26 evaluable patients. Nineteen of these patients were treated with trastuzumab (Herceptin) in combination with a taxane; one received Herceptin in combination with vinorelbine; and six received single-agent Herceptin, without chemotherapy. Figure 1 represents the ROC (receiver operating characteristic) curve for disease progression as a function of the percentage of change in HER2/neu from baseline to 2 to 4 weeks after initiation of therapy. The area under the curve is 0.72 (95% CI 0.52 to 0.92). This value of the area under the curve indicates that the variable (percentage of change in HER2/neu from baseline) discriminates between those patients whose disease progressed and those whose disease did not progress.

Examination of the coordinates of the curve indicates that an optimal cutoff point for the variable is 77%. At this value, the *true-positive fraction *is 72.7%, with a true-negative fraction of 53.3%. In addition, the odds ratio for progression is 1.56, which is a relative maximum at the cutoff.

**Figure 2:**
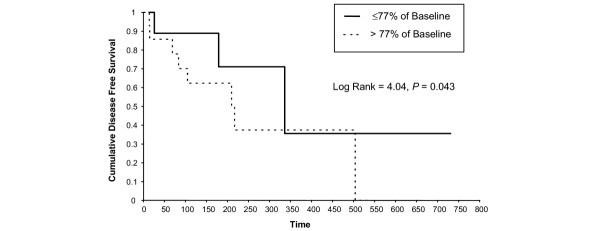
Kaplan–Meier curves for progression-free survival in patients with metastatic breast cancer given trastuzumab-based treatment. Curves were plotted for two groups of patients, those whose HER2/neu concentrations seen at 2 to 4 weeks of trastuzumab-based therapy were ≤ 77% of the baseline concentrations and those for whom it was >77% (median progression-free survival time 587 and 217 days, respectively) *df *= 1. HER, human-epidermal-growth-factor receptor.

Figure 2 shows the Kaplan–Meier curves for progression-free survival in patients with >77% of baseline concentrations of HER2/neu and ≤ 77% of baseline HER2/neu concentrations. The difference between the median progression-free survival times for these two groups, 217 and 587 days, respectively, was significant (log-rank statistic 4.04, *df *= 1, *P *= 0.043).

A subcohort of 52 patients treated specifically with a combination of trastuzumab and taxane was stratified according to the patients' baseline HER2/neu concentrations; 35 had elevated baseline concentrations (≥ 15 ng/mL) and 17 had baseline concentrations <15 ng/mL. Kaplan–Meier analysis for progression-free survival in these two groups showed no significant differences (log-rank statistic = 0.00, *df *= 1, *P *= 0.12).

**Figure 3:**
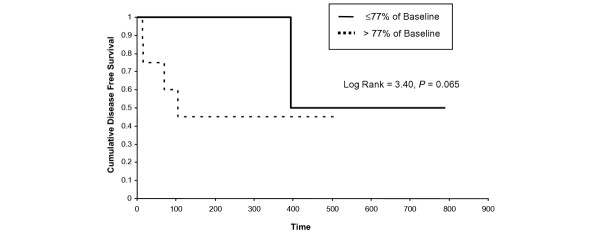
Kaplan–Meier curves for progression-free survival in patients with metastatic breast cancer receiving trastuzumab–taxane. Differences between the survival times (days) for patients whose HER2/neu concentrations at 2 to 2 weeks of treatment had fallen to ≤ 77% of baseline values and those whose values had remained at >77% of baseline values (respective survival times 587 and 119 days).

Additionally, we evaluated, in a subpopulation of 15 patients, the association between percentage of baseline HER2/neu concentrations found 2 to 4 weeks after the start of trastuzumab–taxane therapy and progression-free survival. The median progression-free survival in the group of patients whose HER2/neu value fell to ≤ 77% of baseline (median decrease for the cohort) at 2 to 4 weeks of therapy was 587 days, whereas in the group whose HER2/neu values were >77% of baseline values, the median progression-free survival time was 119 days (Fig. 3). Although there was no significant difference observed in progression-free survival for these two groups of patients (log-rank statistic = 3.40, *df *= 1, *P *= 0.065), there was a trend that was similar to that observed for the larger cohort of patients who were undergoing any of the trastuzumab–based therapies included in our study.

## Discussion

In this cohort, the HER2/neu ECD concentration after 2 to 4 weeks of treatment relative to that at baseline predicted progression-free survival in patients undergoing trastuzumab-based therapy for metastatic breast cancer. Since most patients would not usually undergo imaging studies for assessment of their response to therapy after 2 to 3 months, the serum HER2/neu ECD data may be clinically useful to determine if an individual patient is responding, or whether earlier imaging studies are warranted. Furthermore, in our patients, the serum HER2/neu ECD provided additional value over tumor marker CA15-3.

Currently, patients with metastatic breast cancer are selected for trastuzumab-based therapy if the HER2/neu protein is overexpressed (immunohistochemistry score 3+) in the primary tumor, or if FISH provides evidence of *HER2/neu *gene amplification [14,15,22]. Although the HER2/neu ECD assay is approved by the FDA for monitoring patients undergoing systemic therapy for metastatic breast cancer, the clinical utility of this marker in patients undergoing trastuzumab-based therapy is not well defined. In 2000, the American Society of Clinical Oncology Tumor Markers Expert Panel did not recommend the use of CEA, CA15-3, CA27-29, or HER2/neu ECD to monitor patients with metastatic breast cancer [23]. Although a rising tumor marker may be detected sooner than radiologically visible progression or than symptoms, available data do not indicate that this approach would lead to improved survival in patients with metastatic breast cancer. However, as novel targeted therapies are developed, specific assays such as the HER2/neu ECD may gain clinical utility [24].

At the time of the report of the expert panel of the American Society of Clinical Oncology, there were no published data regarding circulating HER2/neu ECD and response to trastuzumab-based therapy. Since then, several preliminary studies have found that serum HER2/neu followed the course of disease while patients were receiving trastuzumab monoclonal antibody therapy. Esteva and colleagues [25] evaluated serum HER2/neu in 30 women with tissue HER2/neu overexpressing metastatic breast cancer who were undergoing weekly docetaxel and trastuzumab therapy. They found that serum HER2/neu concentrations decreased in 87% of the responding patients, indicating that changes in serum HER2/neu concentrations correlate well with the clinical course of disease. Furthermore, they showed that patients with elevated pretreatment serum concentrations (≥ 15 ng/mL) of HER2/neu had a high (76%) overall response rate to therapy, compared with a 33% response rate in patients with baseline concentrations <15 ng/mL. Dnistrian and collaborators [26] evaluated serum HER2/neu in 54 patients who had metastatic breast cancer with tissue overexpression of HER2/neu and who were undergoing trastuzumab therapy either alone or in combination with paclitaxel. Similar to the results of Esteva and colleagues, they found that among patients with an abnormal pretreatment serum HER2/neu, 83% responded favorably to trastuzumab therapy. Futhermore, Kostler and coworkers [27] evaluated the clinical utility of serum HER2/neu in the early prediction of response to trastuzumab-based therapy in 55 patients with tissue HER2/neu-overexpressing metastatic breast cancer. They found that the ratio of the relative values of HER2/neu from each serial sample during trastuzumab treatment to the baseline HER2/neu concentration before initiation of trastuzumab therapy were significantly decreased in patients responsive to treatment in the early weeks after initiation of therapy. These authors showed that serial HER2/neu concentrations could predict the risk for disease progression as early as day 15 of treatment.

Burstein and collaborators [28] measured HER2/neu ECD concentrations at baseline and during treatment with vinorelbine and trastuzumab. In that study, HER2/neu ECD concentrations were not predictive of response to therapy, but a lack of decline in that concentration was a predictor for tumor progression after cycle 1. In our retrospective study, baseline HER2/neu ECD concentrations were not predictive of response to therapy or time to progression. However, patients who had ≤ 77% of baseline in serum HER2/neu ECD concentrations 2 to 4 weeks after initiation of therapy had a longer time to progression (median 587 days) than patients with >77% of baseline concentrations of HER2/neu ECD (median 217 days). A similar trend in the prediction of progression-free survival was observed in the subcohort of patients treated with a combination of trastuzumab and taxane.

One of the limitations of this study is that we evaluated a selected population, since all patients have some degree of HER2 expression in their primary tumors. However, approximately two-thirds of HER2/neu tissue-positive patients do not respond to single-agent trastuzumab [15], and approximately a third of HER2/neu tissue-positive patients do not respond to trastuzumab in combination with chemotherapy [15,25]. Serum HER2/neu allows for real-time assessment of HER2 status, which could be quantified, and because it is a noninvasive procedure repeated testing is possible to determine the change in HER2/neu concentrations. Therefore, we believe that serum HER2 ECD concentrations in patients with metastatic disease complements the data regarding tissue expression at the time of initial diagnosis (generally, HER2/neu status is assessed in primary breast cancer specimens, not in metastatic deposits).

## Conclusion

This article does not advocate using baseline serum HER2/neu to predict the efficacy of trastuzumab (Herceptin) therapy. However, it does show value in using the delta change in HER2/neu ECD from baseline to 2 to 4 weeks after the start of therapy. Monitoring efficacy during the course of therapy would allow clinicians to make necessary adjustments to drug combination or even prompt them to follow up patients more frequently. Our hypotheses are currently being tested in a prospective, multicenter clinical trial.

## Abbreviations

CA = cancer antigen; ELISA = enzyme-linked immunosorbent assay; ECD = extracellular domain; FISH = fluorescence *in situ *hybridization; FDA = Food and Drug Administration; HER = human-epidermal-growth-factor receptor.

## Competing interests

CC and FG are employees at Bayer Diagnostics. FJE, MF, DS, and DL received research funds from Bayer Diagnostics.

## Authors' contributions

All authors read and approved the final manuscript. FJE, CDC, and FG conceived of the study, participated in its design and coordination, and helped to draft the manuscript. HB, MF, DS, and DL participated in the study design and coordination and helped to draft the manuscript. RPT participated in the design of the study and performed the statistical analysis.

## Figures and Tables

**Table 1:** Characteristics of 103 women with HER2/neu-overexpressing metastatic breast cancer

Patient characteristic	*n*	*%*
HER2/neu overexpression^a^		
Score 2+	31	30.1
Score 3+	72	69.9
Treatment		
Single-agent trastuzumab	13	12.6
Trastuzumab + taxane	52	50.5
Trastuzumab + vinorelbine	11	10.7
Trastuzumab + taxane + megestrol acetate	6	5.8
Trastuzumab + taxane + tamoxifen	5	4.8
Trastuzumab + bevacizumab	5	4.8
Trastuzumab + taxane + carboplatin	4	3.9
Trastuzumab + taxane + CMF	3	2.9
Trastuzumab + erlotinib	2	1.9
Trastuzumab + doxorubicin + cyclophosphamide	2	1.9

^a^Scores indicating HER2/neu overexpression as determined immunohistochemically using the Dako Herceptest. CMF, cyclophosphamide, methotrexate, fluorouracil; HER, human-epidermal-growth-factor receptor.

**Table 2:** Estimates of concordance of serum values with clinical status of patients treated with trastuzumab

Concordance	HER2/neu	CA15-3	Series test^a^
C^b^	0.793	0.627	0.829

^a^Change was considered positive if it was ≥ 15% for HER2/neu *and *≥ 21% for CA15-3. ^b^Differences in concordance for HER2/neu and CA15-3 with 95%CI = 0.166 (lower limit 0.102, upper limit 0.227).
